# The extent of liver injury determines hepatocyte fate toward senescence or cancer

**DOI:** 10.1038/s41419-018-0622-x

**Published:** 2018-05-14

**Authors:** Chao Wang, Wen-Jian Chen, Ying-Fu Wu, Pu You, Shang-Yong Zheng, Chang-Cheng Liu, Dao Xiang, Min-Jun Wang, Yong-Chao Cai, Qing-Hui Zhao, Uyunbilig Borjigin, Wei Liu, Wu-Jun Xiong, Kirk J. Wangensteen, Xin Wang, Zhong-Min Liu, Zhi-Ying He

**Affiliations:** 1 Department of Cell Biology, Center for Stem Cell and Medicine, Second Military Medical University, 200433 Shanghai, China; 2 Institute for Regenerative Medicine, Shanghai East Hospital, School of Life Sciences and Technology, Tongji University, 200123 Shanghai, China; 3 School of Medicine, Tongji University, 200092 Shanghai, China; 4 School of Medicine, Yunnan University, 650091 Kunming, Yunnan China; 5 Naval Medicine Research Institute, Second Military Medical University, 200433 Shanghai, China; 6 The Key Laboratory of National Education Ministry for Mammalian Reproductive Biology and Biotechnology, Inner Mongolia University, 010070 Huhhot, China; 7 Department of Hepatology, Shanghai East Hospital, School of Medicine, Tongji University, 200120 Shanghai, China; 8 Department of Medicine, Division of Gastroenterology, University of Pennsylvania, Philadelphia, PA 19104 USA; 9 Department of Laboratory Medicine and Pathology, University of Minnesota, Minneapolis, MN 55455 USA; 10 Hepatoscience Incorporation, 725 San Aleso Ave, Sunnyvale, CA 94085 USA

## Abstract

It is well known that induction of hepatocyte senescence could inhibit the development of hepatocellular carcinoma (HCC). Until now, it is still unclear how the degree of liver injury dictates hepatocyte senescence and carcinogenesis. In this study, we investigated whether the severity of injury determines cell fate decisions between hepatocyte senescence and carcinogenesis. After testing of different degrees of liver injury, we found that hepatocyte senescence is strongly induced in the setting of severe acute liver injury. Longer-term, moderate liver injury, on the contrary did not result into hepatocyte senescence, but led to a significant incidence of HCC instead. In addition, carcinogenesis was significantly reduced by the induction of severe acute injury after chronic moderate liver injury. Meanwhile, immune surveillance, especially the activations of macrophages, was activated after re-induction of senescence by severe acute liver injury. We conclude that severe acute liver injury leads to hepatocyte senescence along with activating immune surveillance and a low incidence of HCC, whereas chronic moderate injury allows hepatocytes to proliferate rather than to enter into senescence, and correlates with a high incidence of HCC. This study improves our understanding in hepatocyte cell fate decisions and suggests a potential clinical strategy to induce senescence to treat HCC.

## Introduction

Hepatocellular carcinoma (HCC) is one of the most frequent human malignancies with poor prognosis^1^. Nowadays, the most effective treatment for HCC patients is mainly to resect tumors because of the effective drugs deficiency. Only a minority of patients are candidates for surgerical extirpation or transplantation. Thus, a better grasp of the cellular and molecular mechanisms underlying HCC is needed in order to find effective strategies for the treatment of HCC.

The role of cell senescence on inhibition of carcinogenesis has been suggested in several recent studies^2–10^. For example, programmed cellular senescence was found to trigger innate immune responses targeting to liver tumor cells^9^. However, the mechanisms for induction of hepatocyte senescence have not been characterized until now. Here, we characterize the level of injury as a crucial trigger for senescence. Pathological changes of hepatocytes have been described according to the time length and severity extent of different injuries^11^. Remarkably, a high incidence of HCC is known to occur in humans with chronic liver injuries, such as with alcohol addiction, fatty liver disease, viral hepatitis or other chronic liver diseases. Animal models have been able to replicate most of these types of chronic liver injuries to study carcinogenesis. However, up to now, there has been no standard to clearly define how level of liver injury relates to carcinogenesis. Therefore, a better understanding of the inter-relationships among liver injuries, hepatocyte senescence and carcinogenesis will help clarify the pathogenesis of HCC, which could guide therapeutic strategies for HCC.

Patients with hereditary tyrosinemia have been described to undergo fulminant liver failure–leading to death–or more prolonged injury with cirrhosis and greatly increased risk of HCC^12–14^. The mouse model of this condition, the fumarylacetoacetate hydrolase knockout (*Fah*^−/−^) mice, are a robust model for inducible liver injury and liver repopulation, for example with Fah expression vectors^15,16^, or by transplantation of hepatocytes^17–20^. *Fah*^−/−^ mice have been used in several studies on carcinogenesis^18,19^. For example, hepatocytes have been found to be resistant to apoptosis during liver injury in *Fah*^−/−^ mice, which promotes carcinogenesis through activation of the AKT pathway^21^. More recently, a unique role of the adaptive immune system on promoting carcinogenesis was found in *Fah*^−/−^ mice, and resultant HCCs closely matched the genetic profile of HCCs in humans^22^.

Here, we investigated the inter-relationship among the level of liver injury, hepatocyte senescence and carcinogenesis. We discover that the degree of liver injury determines the hepatocyte cell fate into senescence or carcinogenesis, paving a way to potentially treat or prevent HCC by inducing hepatocyte senescence.

## Results

### Induction of hepatocyte senescence by severe acute liver injury

Cellular injuries can result in senescence, apoptosis or necrosis^23^. In *Fah*^−/−^ mice, hepatocytes undergo toxic injury by the abnormal accumulation intermediate metabolites of tyrosine catabolism^24^. The drug 2-(2-nitro-4-trifluoromethylbenzoyl)-1,3-cyclohexanedione (NTBC) specifically blocks toxin formation by inhibiting an enzyme upstream of FAH in the tyrosine metabolic pathway. Daily administration of therapeutic doses of NTBC restores liver function and keeps patients with hereditary tyrosinemia and *Fah*^−/−^ mice completely healthy^24–26^. In *Fah*^−/−^ mice, total withdrawal of NTBC has been shown to induce severe acute liver injury (SALI), and lead to death within 6–8 weeks, while a reduction of NTBC amount to 2.5% of the therapeutic dose induces moderate chronic liver injury (MCLI)^27^.

In order to characterize the relationship between the level of injury and senescence in hepatocytes, we first examined the histopathology of *Fah*^−/−^ mouse livers after NTBC withdrawal. As seen with severe acute hepatic injuries in humans, with SALI we detected abundant hepatocyte multinucleation, bile duct hyperplasia, severe hepatocyte steatosis and swelling (Fig. S1A-C). On the other hand, with MCLI, milder pathological changes were detected, including micro-inflammation, foci of necrosis and micro-steatosis, slight nuclear polymorphism and hepatocyte swelling (Fig. S1A-C). In addition, analyses on serum indexes of liver function were used to confirm the degrees of liver injury in SALI and MCLI mice. We found that the serum indexes of ALT, AST, and T-bil were significantly higher in the SALI group than the MCLI group (Fig. S2A). There was a significant increase in cell size with 4 weeks SALI (Fig. S1A, D, F), which is generally regarded as indicative of cellular senescence^28^. In contrast, no obvious enlargement occurred in MCLI (Fig. S1A, E, F). TUNEL-positive cells were not detectable in either SALI or MCLI (Fig. S2B, C), which suggested that hepatocytes were apoptosis-resistant, as reported previously^27,29,30^.

**Fig. 1:**
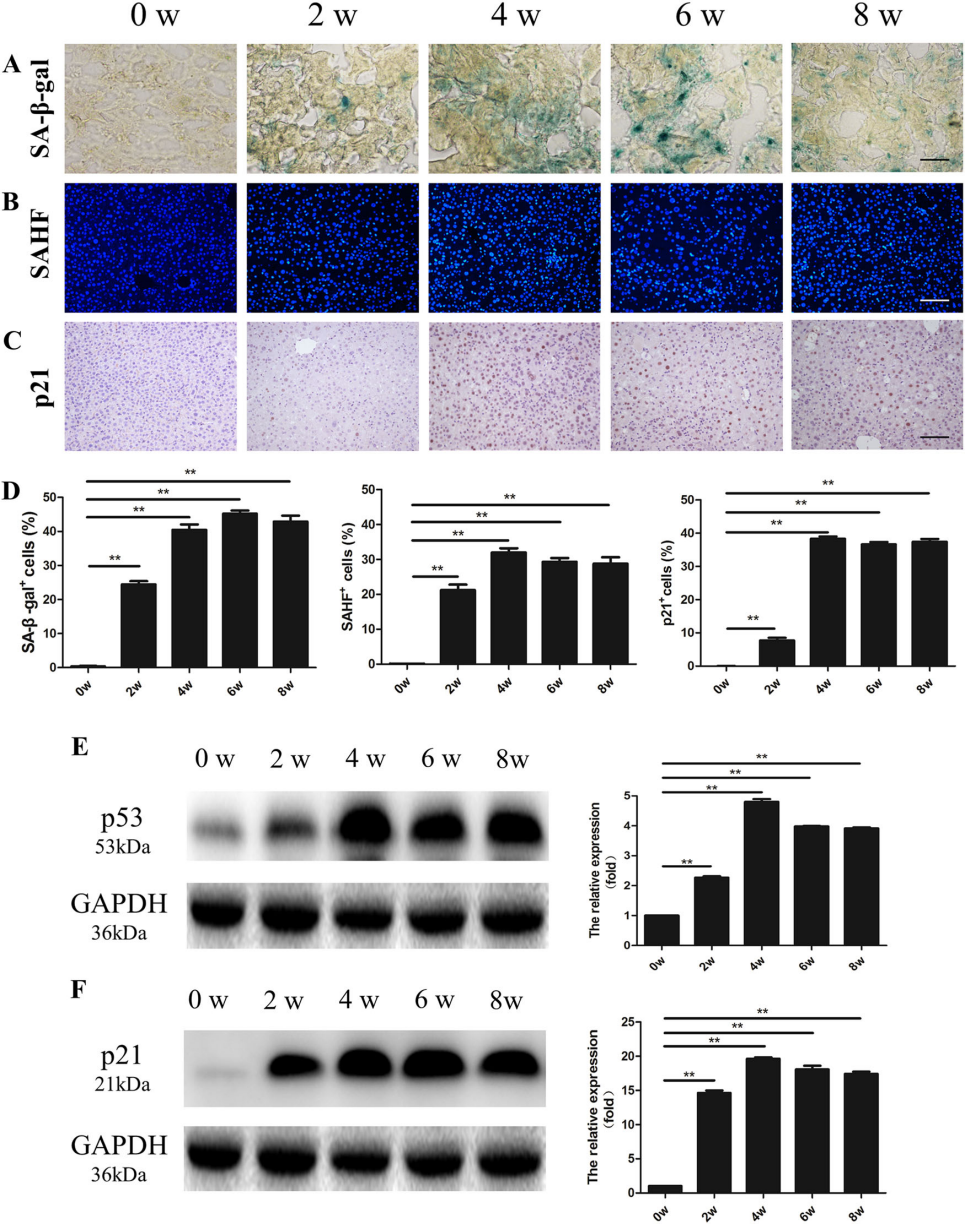
Severe acute liver injury leads to hepatocyte senescence. **a** Representative images of SA-β-gal activity in the livers of *Fah*^−/−^mice undergoing severe acute liver injury for 0, 2, 4, 6, and 8 weeks. **b** Representative images of SAHF staining in liver sections at 0, 2, 4, 6, and 8 weeks. **c** Immunohistochemistry staining for p21 at 0, 2, 4, 6, and 8 weeks. **d** The bar graphs show the quantification of positive cells for SA-β-gal, SAHF, and p21. **e**, **f** Protein levels of p53 and p21 in livers at 0, 2, 4, 6, and 8 weeks of severe injury. Quantitation of protein levels is shown on the right panel. The baseline value was set on the expression level for *Fah*^−/−^ mice on NTBC and considered equal to 1. All values presented as mean ± S.D. ** *p* *<* 0.01. Scale bar, 100 µm

As apoptosis and necrosis were not evident in either injury condition, we further characterized whether senescence is the major event in SALI in *Fah*^−/−^ mice after total NTBC withdrawal. We examined liver samples at 0, 2, 4, 6, and 8 weeks of treatment for markers of hepatocyte senescence, as in previous studies^31^. Senescence associated-β-galactosidase (SA-β-Gal) activity^32,33^, was undetectable in control tissue, but became positive in nearly 50% of hepatocytes by 8 weeks of SALI (**p *<* 0.01 vs. control, Fig. 1a,d). Similarly, the senescence associated heterochromatin (SAHF) assay^34^ also increased from undetectable levels to more than 30% positive with SALI (**p *<* 0.01 vs. control, Fig. 1b,d). The cell cycle inhibitor p21 staining became positive in hepatocytes only with SALI (**p *<* 0.01, Fig. 1c, d). In fact, the cell cycle inhibitors p53 and p21^35–37^ both increased in expression with SALI (**p *<* 0.01, Fig. 1e,f).

Taken together, the above results indicated that hepatocytes became senescent during SALI.

### Hepatocyte senescence was not significantly induced by moderate chronic liver injury

**Fig. 2:**
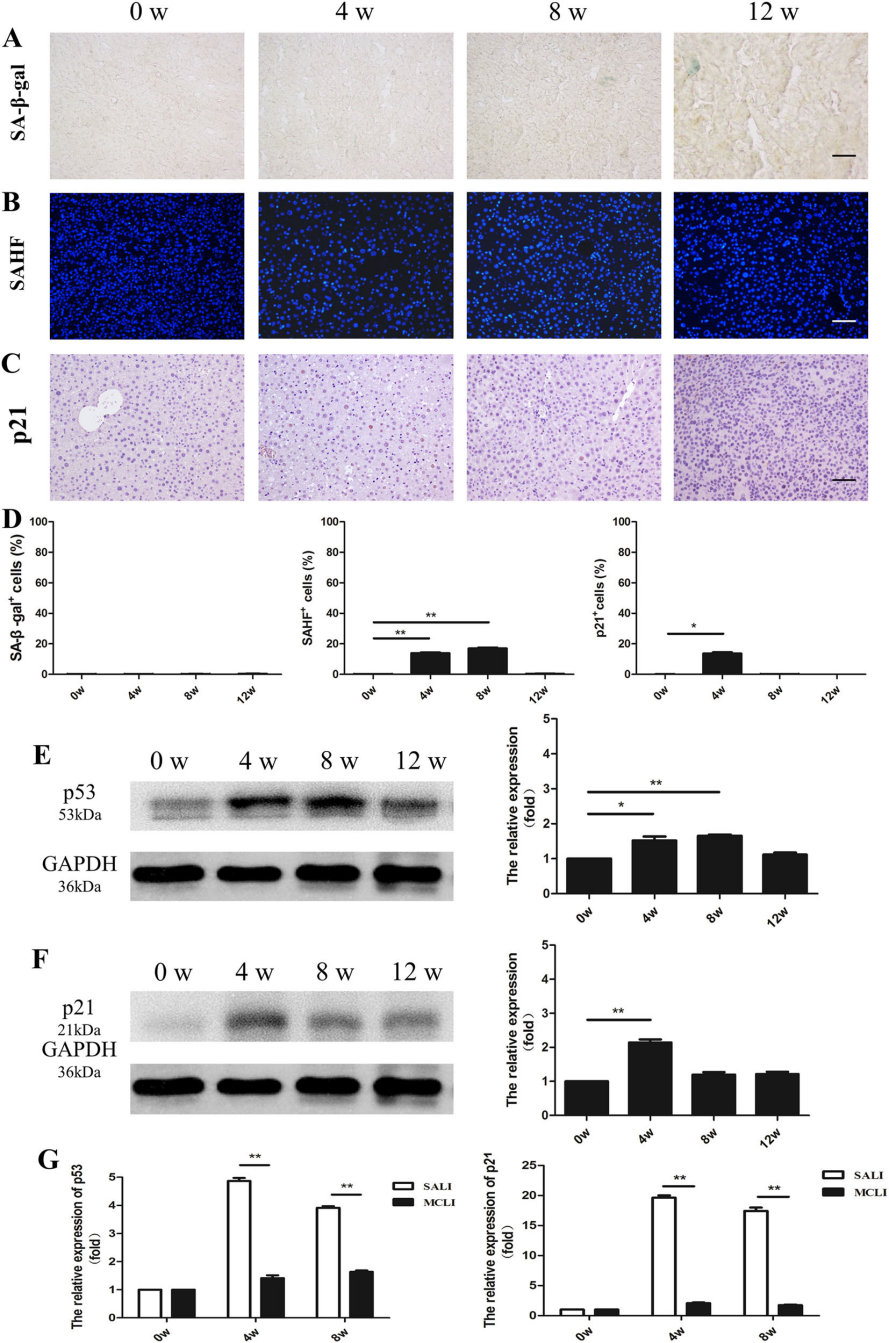
No significant induction of hepatocyte senescence with moderate chronic liver injury. **a** Detection of SA-β-gal activity in the livers of *Fah*^−/−^ mice after reduction of NTBC to 2.5% for 0, 4, 8, and 12 weeks. **b** Staining for SAHF at 0, 4, 8, and 12 weeks. **c** Immunohistochemistry staining for p21 at 0, 4, 8, and 12 weeks. **d** Graph of the number of cells with positive for SA-β-gal, SAHF, and p21. **e**, **f** p53 and p21 expression levels in liver tissue of *Fah*^−/−^ mice in chronic liver injury at 0, 4, 8, and 12 weeks. Quantitation of protein levels was on the right panel. **g** Expression levels of p53 and p21 in livers of *Fah*^−/−^ mice under SALI or MCLI at 0, 4, and 8 weeks. Shows are mean ± S.D. **p *<* 0.01. *p *<* 0.05. Scale bar, 100 µm

Next, we examined whether hepatocyte senescence was induced with MCLI, wherein the NTBC provided to *Fah*^−/−^ mice is reduced to 2.5% of the standard dose^27^. We repeated the assays for cellular senescence during 12 weeks of MCLI. Results indicated that only very rare SA-β-gal-positive cells were detected with up to 12 weeks of MCLI (Fig. 2a,d). Although small proportion (<20%) of cells became positive for either SAHF or p21 at 4 or 8 weeks, these levels decreased to fewer than 5% by 12 weeks (Fig. 2b–d). Western Blotting indicated that the expressions of both p21 and p53 increased at 4 weeks, and then started to decrease significantly from 8 to12 weeks during MCLI (Fig. 2e,f). There were slight increases in expression levels of p53 and p21 with MCLI, generally reaching less than 2-fold, which contrasted with the dramatic increases seen with SALI of more than 4-fold and 15-fold, respectively (Fig. 2g).

In summary, there was little, if any, induction of hepatocyte senescence with MCLI for the 12 week duration of the study.

### Carcinogenesis is strongly induced by MCLI but not by SALI

Previous studies indicated that carcinogenesis is induced with MCLI in *Fah*^−/−^ mice^27,29^. Also, *Fah*^−/−^ mice are known to all die after removal of NTBC for more than 6–8 weeks^24^, indicating that with SALI the animals may die before carcinogenesis is possibly induced. To determine whether carcinogenesis could be induced with longer exposure to SALI, we cycled the NTBC-off periods, so that the mice could survive SALI treatments. Thus, the total time-length of SALI treatment-time was summed up to be equivalent to total time-length of MCLI treatments. Results of both weight curves of *Fah*^−/−^ mice during MCLI or prolonged SALI are summarized in Supplementary Figure 3.

**Fig. 3:**
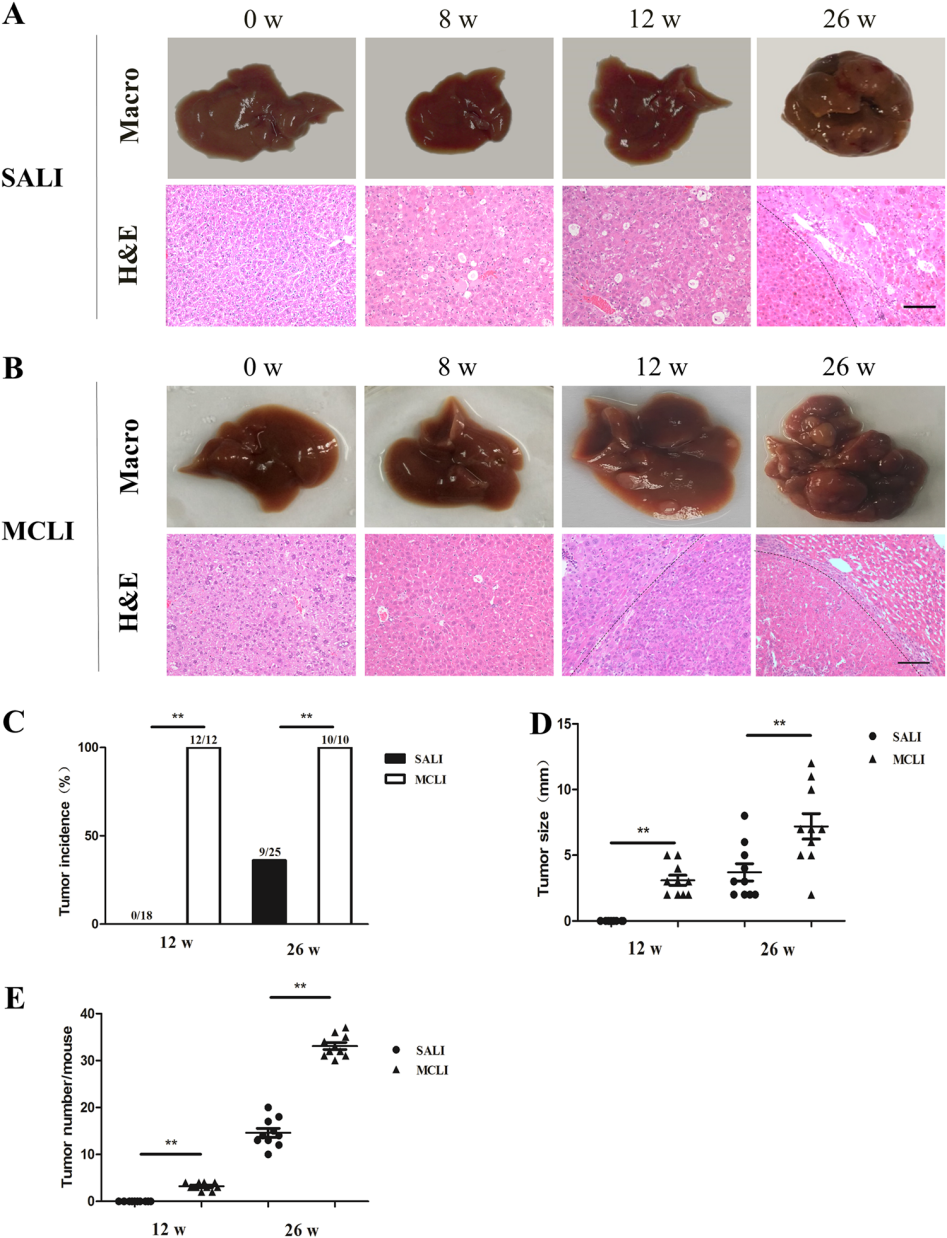
Incidence of carcinogenesis with severe acute and moderate chronic liver injury. **a**, **b** Representative images of livers at 0, 8, 12, and 26 weeks. H&E staining of liver tissues under severe acute or moderate chronic liver injury. **c** Graphs representing tumor incidence at different time points. Interestingly, the incidence of tumor in group SALI was significantly lower than that in group MCLI at 12 and 26 weeks. **d**, **e** Scatter plots displaying the size of tumors (**d**) and tumor numbers (**e**) in the livers at the indicated time points. **p *<* 0.01. Scale bar, 100 µm

The results indicated that no tumor formed after 12 weeks of total SALI treatment-time (Fig. 3a,c; *n* = 18). At 26 weeks of total SALI treatment-time, tumors were found in 9 of 25 mice (Fig. 3a,c; *n* = 25). Each of the 9 mice with tumors had 10 to 20 individual tumor lesions in their livers, ranging in size between 2 to 8 mm in diameter (Fig. 3d,e). On H&E the tumors were found to be HCC (Fig. 3a).

In contrast to SALI, all of the mice undergoing MCLI developed 2 or 3 tumor lesions after 12 weeks, ranging in size from 2 to 5 mm in diameter (Fig. 3b–e; *n* = 12). At 26 weeks MCLI, the numbers of tumor lesions reached to more than 30 per liver of all mice (Fig. 3e). The size of tumor lesions averaged 2 to 12 mm in diameter (Fig. 3d). The tumors were found to be HCC on analysis of H&E sections (Fig. 3b).

In summary, carcinogenesis was strongly induced by MCLI, and was induced with SALI at a reduced rate only with multiple cycles of injury.

### Reduction in carcinogenesis by induction of hepatocyte senescence

**Fig. 4:**
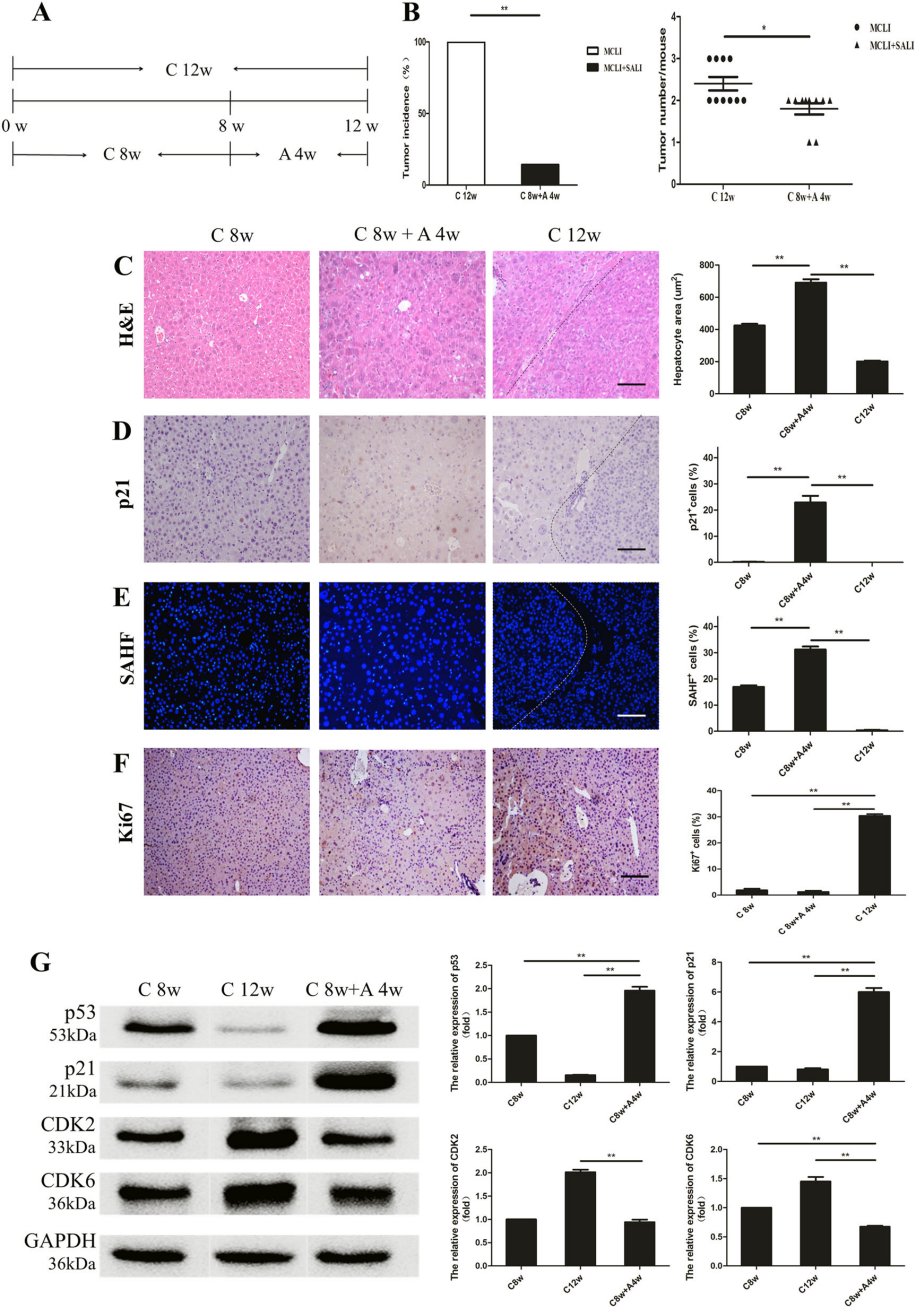
Induction of hepatocytes senescence reduces the occurrence of HCC. **a** Moderate chronic liver injury for 12 weeks (C 12w): *Fah*^−/−^ mice were given 2.5% NTBC for 12 weeks. Moderate chronic liver injury for 8 weeks, followed by severe acute liver injury for 4 weeks (C 8w + A 4w): *Fah*^−/−^ mice were given 2.5% NTBC for 8 weeks, and then treated with SALI for 4 weeks. Livers were collected at 12 weeks. **b** Graphs representing tumor incidence and tumor numbers in C 12w group and C 8w + A 4w group. The incidence of tumor in group C 8w + A 4w was significantly lower than that in group C 12w at 12 weeks. **c**–**e** Indicators of hepatocytes senescence were detected in livers of *Fah*^−/−^ mice after induction of SALI. Hepatocytes size was detected by H&E staining (**c**). p21 (**d**), SAHF (**e**), and Ki67 (**f**) staining were detected by immunohistochemistry. The bar graph on the right is a statistical chart of the corresponding positive cells. **g** Proteins of p53-p21 signaling pathway were analyzed by Western blot. The bar chart of quantitative analysis is on the right. Shows are mean ± S.D. **p *<* 0.01. *p *<* 0.05. Scale bar, 100 µm

Based on the above results, we hypothesized that induction of hepatocyte senescence could reduce the incidence of HCCs in mice undergoing MCLI. As shown above, all mice undergoing MCLI developed HCC by 12 weeks of treatment. Thus, we began 4 weeks of SALI treatment in mice after 8 weeks of MCLI treatment (C 8w + A 4w group), and compared these mice to 12 weeks of continuous MCLI (C 12w group) (Fig. 4a). At 8 weeks, the livers demonstrated moderate, chronic injuries with MCLI treatment (Fig. S1A). We found a marked reduction in tumor incidence from 100% in the C 12w group to less than 15% in the C 8w + A 4w group (**p *<* 0.01, Fig. 4b). Similarly, the number of tumor lesions per mouse was also remarkably reduced in C 8w + A 4w group as compared with C 12w group (Fig. 4b).

We detected the change of hepatocytes in the livers with severe, acute injury in C 8w + A 4w mice. Hepatocyte size was significantly increased in the C 8w + A 4w group when compared to either the C 12w group or C 8w group with MCLI treatment (**p *<* 0.01, Fig. 4c). The number of p21-positive hepatocytes in C 8w + A 4w group was significantly higher than that in both C 12w group and C 8w group (**p *<* 0.01, Fig. 4d). In addition, the number of SAHF-positive hepatocytes was higher in the C 8w + A 4w group than that in both C 8w group and C 12w group (**p *<* 0.01, Fig. 4e). Consistently, the number of Ki67-positive hepatocytes decreased in the C 8w + A 4w group than that in C 12w group (**p *<* 0.01, Fig. 4f). Western blotting confirmed that p53 and p21 expression levels were increased more than 12-fold and 7-fold, respectively, in the C 8w + A 4w group as compared to the C 12w group (Fig. 4g). On the other hand, the expression levels of the cell cycle proteins CDK2 and CDK6 decreased during the same processes (Fig. 4g). Therefore, hepatocyte senescence was strongly induced by SALI after 8 weeks of MCLI.

In summary, the carcinogenesis was significantly reduced by inducing hepatocyte senescence after MCLI. Without the induction of senescence, all of these MCLI pre-treated livers developed HCCs.

### Extensive shift in transcriptome profiles after induction of hepatocyte senescence

**Fig. 5:**
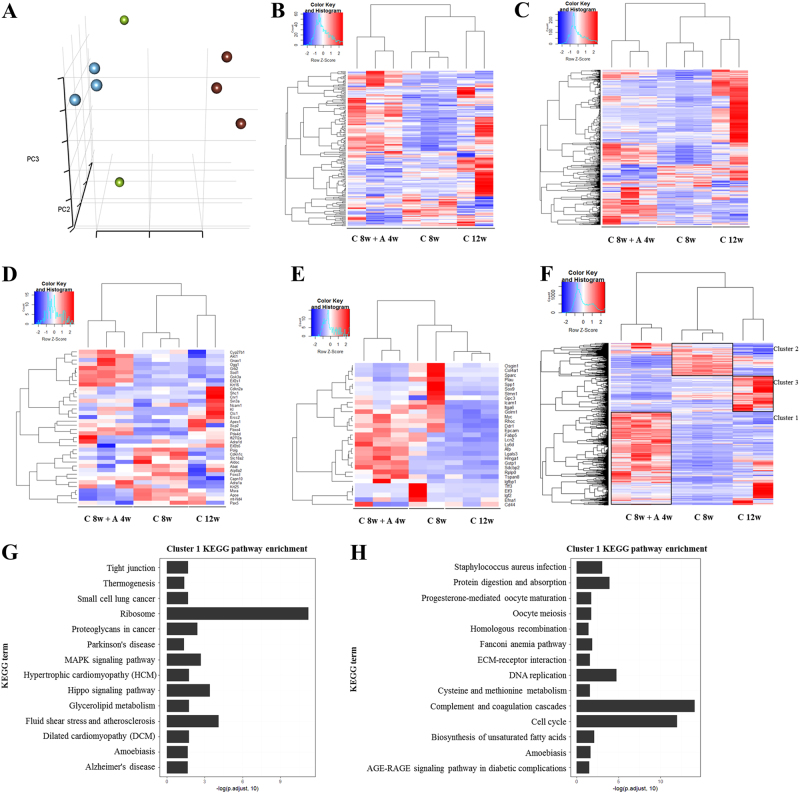
Analysis of gene expression profiles after induction of hepatocyte senescence. **a** Representative results of 3D-PCA for all individual tissue samples from three experimental groups (C 8w, C 8w + A 4w, C 12w). Tissue clusters were differentially colored as each distinct treatment. **b–e** Heatmaps of expression level for genes associated with the Gene Ontology “regulation of cell proliferation”, “cell cycle”, “aging” and “hepatocellular carcinoma”. Analysis of heatmap was obtained after filtering for genes expressed over 10 counts per gene in at least 4 individual samples. Expression values were scaled by using scale function in R. **f** A heatmap built with differentially expressing genes between groups, frames were used to label the three cluster genes. **g**, **h** KEGG enrichment analyses of Cluster 1 and Cluster 3 genes. The abscissa is log10 of adjusted *p*-values

Next, we analyzed the relationships among individual animals from three experimental groups (C 8w, C 8w + A 4w and C 12w) based on their global gene expression patterns. 9980 genes were filtered by using gene and sample requirement-filter function. Principal component analysis (PCA) was then used to visualize the relationships among individual samples from these three groups (Fig. 5a). In the PCA plot, the expression profile of individual samples in C 8w + A 4w group was dramatically separated from the other two groups. Interestingly, individual samples in either C 8w + A 4w group or C 8w group were close to other samples in the same group. On the other hand, individual samples in C 12w group were significantly different for each other. This might be due to the heterogeneity of hepatocellular carcinoma. The results were consistent with previous research that substantial variability was observed among the 373 HCC tumor samples, indicating the presence of tumor heterogeneity^38^.

We also built up heatmaps for genes annotated different Gene Ontology terms about aging, cell proliferation or cell cycle, or for genes known as hepatocellular carcinoma markers using R package gplots. Similarly, all these heatmaps showed that the gene expression profiles in C 8w + A 4w group were separated with which in C 8w and C 12w, and the profilers in C 8w and C 12w were clustered to each other (Fig. 5b-e). These results showed that the transcriptome profiles were globally shifted to another direction by induction of hepatocyte senescence after MCLI.

In order to realize the details of transcriptome profiles, all differentially expressed genes were further clustered, and total of 3 clusters were identified. Each of those clusters mainly expressed in one specific group, respectively (Fig. 5f). After KEGG enrichment analysis, we found that Hippo signaling pathway relative genes were enriched in cluster 1 genes (mainly expressed in C 8w + C 4w group) and Cell Cycle related genes were enriched in cluster 3 genes (mainly expressed in C 12w group) (Fig. 5g,h). Recently, activations of Hippo signaling pathway genes were found to involve into M1-like macrophage recruitment for inhibitions on hepatocellular carcinoma metastasis^39^. To investigate whether macrophage relative genes were also regulated in cluster 1 genes significantly, GO term enrichment analysis was applied in our samples. Results indicated that genes related to macrophage activations were significantly enriched in cluster 1 (*p* *<* 0.05; Supplementary Table 2).

Together, results of transcriptome profiles proved that different degrees of liver injuries induced the hepatocytes to differently enter into either senescence or continuous cell proliferations under particular degrees of liver injuries. Importantly, a new hypothesis could be suggested, which is that macrophages could be activated after re-induction of senescence.

### Senescence inhibits carcinogenesis by activating immune surveillance

Recently, cell senescence induced-immune surveillance has been found to be a critical mechanism inhibiting carcinogenesis^10,40,41^. Here, we clarified whether this is the mechanism for inhibiting carcinogenesis in SALI. We investigated whether senescent hepatocytes could enhance the proliferation of immune cells, especially the activations and proliferations of macrophages, for removing the pre-malignant senescent cells^10^.

**Fig. 6:**
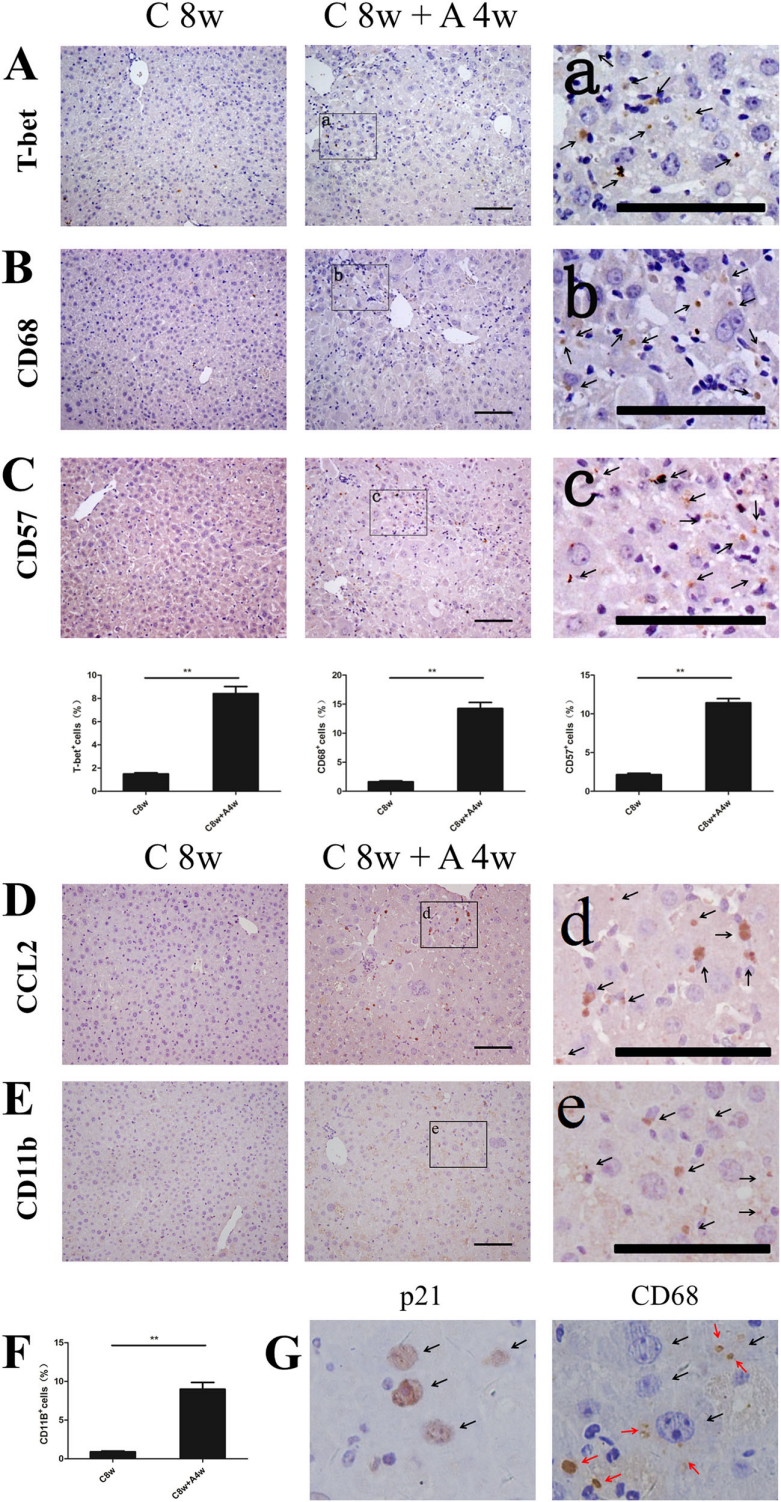
Activation of immune surveillance upon induction of senescence. **a** CD4^+^ Th1 cells were detected by staining for T-bet. **b** Staining for macrophages in the livers with IHC for CD68. **c** Staining for NK cells with IHC for CD57. **d** The bar charts show the positive rate of CD4^+^ Th1 cells, macrophages, and NK cells in the two groups (C 8w and “C 8w + A 4w”). **e** Staining for chemokine CCL2. **f** CD11b staining for senescence-induced myeloid cells. **a–e** Representative photographs are the enlarged views of the corresponding position. **f** The bar chart shows the positive rate of senescence-induced myeloid cells in the two groups (C 8w and “C 8w + A 4w”). **g** Serial sections with staining of p21 and CD68 for senescent hepatocytes and macrophages. Black arrows indicate the senescent cells, and red arrows indicate the macrophages. Shows are mean ± S.D. **p *<* 0.01. Scale bar, 100 µm

We performed T-bet staining, which revealed that the percentage of CD4^+^ Th1 cells of livers in C 8w + A 4w group was significant higher than that in C 8w group (8.41 ± 2.38% vs.1.50 ± 0.34%; Fig. 6a; **p *<* 0.01). Similarly the percentage of CD68 positive macrophages was also significantly higher in the C 8w + A 4w group than that in the C 8w group (14.26 ± 3.98% vs.1.64 ± 0.57%; Fig. 6b; **p *<* 0.01). CD57-positive NK cells were also found increased in the C 8w + A 4w group vs. the C 8w group (9.37 ± 4.85% vs. 3.36 ± 0.59%; Fig. 6c; **p *<* 0.01). Together, these results suggested that senescent hepatocytes could enhance the activation of immune cells. Meanwhile, we also found that the level of macrophages in C 8w + A 4w group were 8.69 times higher than that in C 8w group, while the level of CD4^+^ Th1 cells and NK cells were 5.61 times and 2.79 times higher in C 8w + A 4w group than C 8w group.

Recently, it was known that chemokine CCL2 (MCP-1) secreted from senescent hepatocytes recruited CCR2^+^ immature myeloid cells (iMC) for differentiations into macrophages to execute the clearance of pre-malignant senescent cells^10^. Our results from bioinformatics analyses (Fig. 5f,g and Supplementary Table 2) suggested that the scavenging effect of macrophages might also play an important role during the process of inhibitions on carcinogenesis in the re-induction of senescence group. We found that CCL2 had much higher expression in C 8w + A 4w group than C 8w group (Fig. 6d). Besides of the increased expression of CCL2, CD11b-positive senescence-induced myeloid cells were also increased in the C 8w + A 4w group vs. the C 8w group (8.98 ± 3.41% vs. 0.87 ± 0.34%; Fig. 6e,f; **p < 0.01). Therefore, CCL2 could enhance both recruitment and maturation of senescence-induced myeloid cells. In addition, the activated macrophages were found to spread around the senescent hepatocytes in livers of C 8w + A 4w group (Fig. 6g), which supported our hypothesis that senescence could inhibit carcinogenesis by the activating immune surveillance initiated by macrophages.

## Discussion

Here, our study indicates that the severe liver injury leads hepatocytes to enter senescence. With reduced injury, the hepatocytes do not enter senescence, and are more likely to develop HCC. Our present findings suggest that for hepatocytes to enter senescence they require a threshold of liver injury.

The recent discovery on relationship between hepatocyte senescence and inhibition of carcinogenesis opens our scope to realize the underlying mechanisms of carcinogenesis in HCC, and opens the door to new treatment possibilities^10^. Re-induction of hepatocyte senescence by SALI treatment could inhibit carcinogenesis through the activation of immune surveillance. It is also possible that hepatocyte senescence by SALI treatment could induce other unknown mechanisms to inhibit carcinogenesis. The results of our present study support the development of strategies to induce senescence to treat or prevent HCC.

Different from previous reports on the induction of hepatocyte senescence to inhibit carcinogenesis^41^, our findings were achieved by using an animal model that is closely mimics clinically relevant liver disease^24^. In *Fah*^−/−^ mice, the inactivation of Fah leads to an accumulation of toxic metabolites, such as fumarylacetoacetate (FAA), which damages DNA and subsequently causes SALI^30^. *Fah*^−/−^ mice generally die of acute liver injury 4–8 weeks after removal of NTBC. *Fah*^−/−^ mice also are very useful for studying carcinogenesis by integrating various genetic backgrounds, such as the p21-null liver^29^, or by expression of oncogenes^15,42,43^, or by modulating the level of injury or cycling NTBC^29^.

In some previous studies of carcinogenesis, *Fah*^−/−^ mice were all analyzed under situation of MCLI^29^. MCLI is usually a complex pathological process typically characterized by the continuous low-level hepatocyte destruction and renewal, which mimics alcohol abuse, drug toxicity, viral infection, and genetic metabolic disorders. In the present study, we control the degree of liver injury in *Fah*^−/−^ mice, and we were able to precisely evaluate the differences in the hepatocytes for choosing dual fates. Therefore, the degree of liver injury controls the initiation of the activations on signaling pathways specific to either carcinogenesis or hepatocyte senescence. With the in-depth researches on downstream activities after SALI or MCLI, the potential key factors to switch to either hepatocyte senescence or carcinogenesis could be realized, which will be helpful for design clinical protocols of HCC therapy. Our results also indicate that re-induction of senescence could activate immune surveillance to inhibit carcinogenesis, which further supported our hypothesis on SALI inducing hepatocyte senescence to inhibit carcinogenesis. The senescent hepatocytes can enhance the activations of immune cells, including macrophages, CD4^+^ Th1 cells and NK cells, etc. Our results indicate that macrophage activation may play a crucial role in the process of senescence surveillance^10,40,41^. As with previously characterized mechanism, our results suggest that SALI induces hepatocyte senescence, promotes activation and proliferation of macrophages, and then induces cytotoxic scavenging and removal of pre-malignant senescent hepatocytes to suppress carcinogenesis. However, existences of other unknown mechanisms are still possible during future studies.

**Fig. 7:**
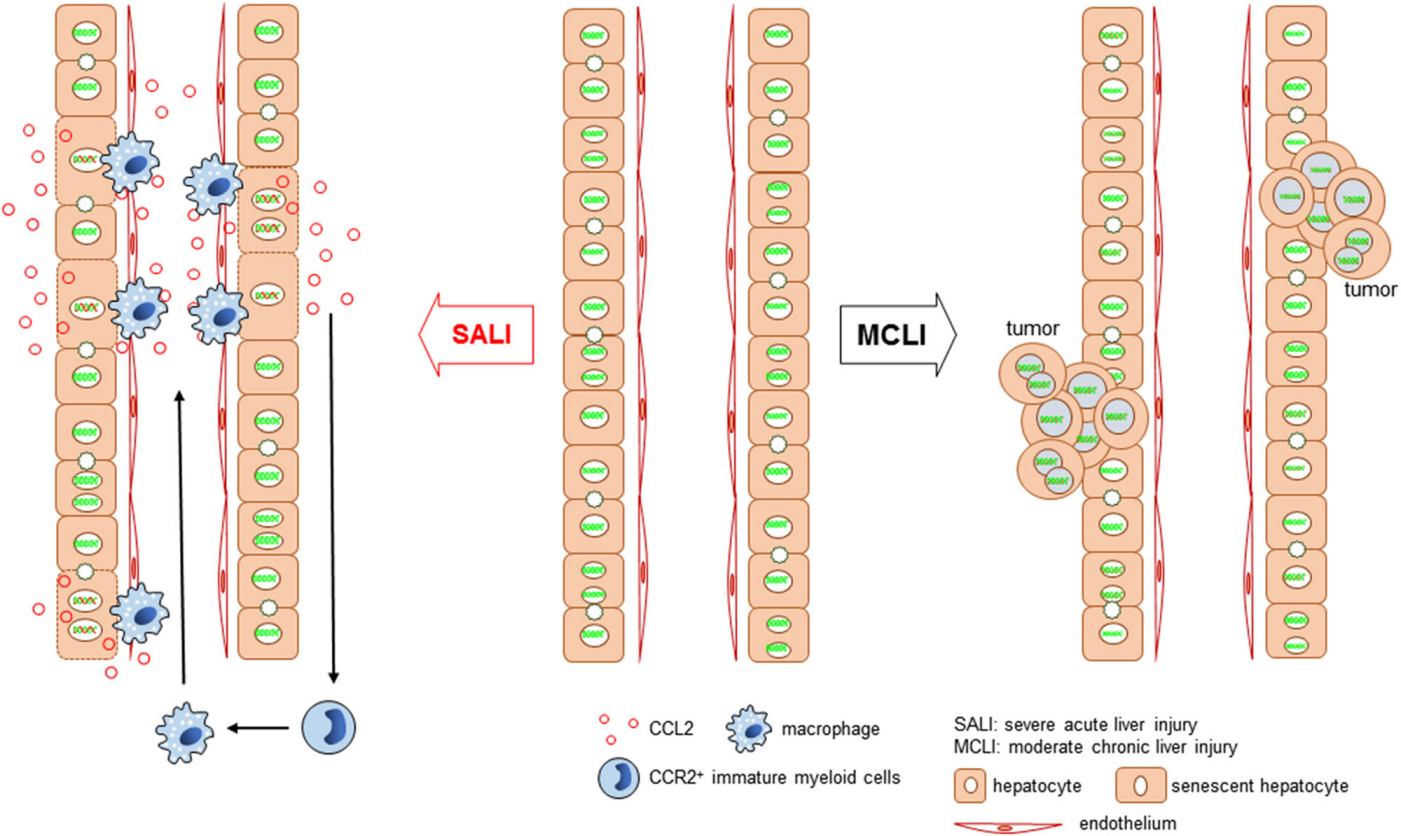
Schematic diagram of the relationship between hepatocyte senescence and carcinogenesis during liver injury

For the first time, our research indicates the existences of a special inter-relationship between liver injury and hepatocyte senescence, as well as liver injury and carcinogenesis during different inductions on degrees and time lengths of liver injury in vivo. A schematic diagram for such relationships was summarized in Fig. 7. Hepatocyte senescence as a protective mechanism for inhibition carcinogenesis could be useful for the therapies on HCC in the future.

## Materials and methods

### Mice

All the experimental mice were the same strain (129S4 background). All mice received humane care according to the guidelines of Second Military Medical University Animal Care and Use Committees.

### Severe acute and moderate chronic liver injury models

*Fah*^−/−^ mice were maintained on continuous NTBC in their drinking water (7.5 mg/L). In severe acute liver injury (SALI), NTBC was totally withdrawn. In order to keep *Fah*^−/−^ mice survive under prolonged, SALI treatments were divided into several stages. For each stage intervals between two SALI treatments, the *Fah*^−/−^ mice were taken back to standard administration of NTBC for three days. Using this method let the total time-length of collective treatments of SALI equal to total time-length of CLI treatments. Mice exposed to a reduced treatment regimen of NTBC (2.5%) in the moderate chronic liver injury (MCLI) group. In the induced senescence group (MCLI8 + SALI4), *Fah*^−/−^ mice were given 2.5% curative dosage NTBC for 8 weeks, and then NTBC was removed for 4 weeks.

### Hematoxylin-Eosin staining (H&E), Immunohistochemistry (IHC), Oil red O staining, and Serum indexes

For hematoxylin-eosin staining, fresh liver tissues were fixed with 4% paraformaldehyde (PFA), then were routinely embedded in paraffin and sectioned into slices (2 µm). The slices were processed for roasting, dewaxing and rehydration, and were stained with hematoxylin (Beyotime, Shanghai, CHN) for 5–10 min, rinsed with water for 15 min, sliced into 95% alcohol (Sinoreagent, Shanghai, CHN) for 30 seconds, and then stained with eosin (Beyotime, Shanghai, CHN) for an appropriate amount of time (0.5–2 min). Finally, the slice is dehydrated rapidly and mounted with neutral resin (MXB, Fujian, CHN).

For immunohistochemistry staining, IHC steps are the same as H&E before rehydration. The slices were soaked in 0.01 M citric acid buffer (pH 6.0) and placed in a pressure cooker for 2–4 min at 121 °C/100 kpa. Cool to room temperature. 3% H_2_O_2_ solution blocked endogenous peroxidase and 1% BSA blocked nonspecific loci for 30 min at room temperature. The slices were incubated with primary antibodies for at 4 °C overnight and secondary antibodies conjugated with HRP at 37 °C for 30 min. Staining with DAB (Vector Laboratories, Burlingame, CA) was applied to the sections. The sections were stained with hematoxylin (Beyotime, Shanghai, CHN), dehydrated rapidly and mounted with neutral resin (MXB, Fujian, CHN). The list of primary and secondary antibodies is summarized as Supplementary Table 1.

For Oil red O staining, freshly harvested liver tissues were embedded in the optimum cutting temperature compound (Sakura Finetek, Torrance, CA). The tissues was frozen and sliced to sections with thickness of 10 µm. Staining of the slices was according to previously established protocol of the Oil red O staining. Finally, the sections were stained with hematoxylin (Beyotime, Shanghai, CHN) for 5–10 min, rinsed with water for 15 min.

For Serum indexes, sample blood was collected from the retro-orbital sinus of test animals. Plasma was prepared using Microtainer plasma separator tubes (BD) and stored at −80 °C. Analysis the biochemical indexes of serum was according to the previously established protocol.

### Transferase-mediated deoxyuridine triphosphate-biotin nick end labeling (TUNEL) staining

The staining is carried out according to the kit instruction (Beyotime Biotechnology). TUNEL staining steps are the same as H&E before rehydration. The slices were treated with protease K at room temperature for 15–30 min. After PBS washing, 3% H_2_O_2_ solution blocked endogenous peroxidase. Biotin-dUTP solution was incubated at 37 ℃ for 60 min and the elimination agent was dripped. Finally, Streptavidin-HRP working solution is added to the section at room temperature for 30 min. Staining with DAB (Vector Laboratories, Burlingame, CA) was applied to the sections.

### Senescence associated-β-galactosidase (SA-β-Gal) activity

The staining is carried out according to the kit instruction (Beyotime Biotechnology). Fresh tissue was embedded in optimum cutting temperature compound (Sakura Finetek, Torrance, CA). The tissue was frozen and cut into slices of 5–7 µm. Add proper volume of SA-β-Gal staining fixative solution to the section and fix it at room temperature for 15 min. Finally, the sections were incubated in fresh SA-β-Gal stain solution at 37 °C.

### Protein isolation and Western blot

100× protease inhibitors and 100× PMSF were added into the lysate. Appropriate amount of cell lysate (Invent Biotechnologies, Eden Prairie, MN) was added to the liver tissue. The tissue was cut and homogenized, and then it was incubated on ice for 30 min. Centrifuge at 4 °C, 12,000 r.p.m. 10 min. Aspirate the supernatant and place in a fresh tube kept on ice, and discard the pellet. Add 5× loading buffer, and boiled at 100 °C for 3–5 min, store at −20 °C or −80 °C. Separate proteins from sample lysates using a standard sodium dodecyl sulfate polyacrylamide gel electrophoresis (SDS-PAGE) protocol. Transfer proteins from the gel to a PVDF membrane (Millipore, Temecula, CA), 350 mA, 90 min. Block the membrane using a solution of 5% non-fat dry milk in TBST for at least 60 min, RT. Incubate the membrane with primary antibody (diluted in TBST) at 4 °C overnight. Wash the membrane three times for 5 min each in TBST. Incubate the membrane with secondary IgG Peroxidase conjugate. Protein bands were detected by chemiluminescent substrate (Thermo Scientific, Waltham, MA).

### RNA sequencing and bioinformatics analysis

RNA sequencing technology is provided by Shanghai OE Biotech Co., Ltd. PCA and hierarchical clustering were performed using R^44^. PCA was used on gene and sample requirement-filtered^45^ (any gene with reads less than 10 and with samples less than 4 has been removed) data to visualize differences between groups. Hierarchical cluster analysis on a set of dissimilarities and “complete” method for analyzing different genes based on the same gene ontology (i.e., “regulation of cell proliferation”, “cell cycle” or “aging”) or “hepatocellular carcinoma” marker genes^46^. The GOseq R package was used to perform GO enrichment analysis of different cluster genes. A corrected *P*-value < 0.05 was adopted as the standard for judging statistically significant enrichment of cluster genes. KEGG enrichment analysis of different cluster genes was implemented using clusterProfiler R package, and the cutoff for P-value was set at 0.05. Raw sequencing data is publically available at NCBI (GEO accession number GSE108328).

### Statistical analysis

All data are presented as mean ± standard deviation (S.D.). Experimental data were analyzed by a Student *t* test with Statistical Program for Social Sciences software (IBM SPSS, IBM Corporation, Somers, NY, USA) to determine significance. *p* < 0.05 was considered statistically significant.

## Electronic supplementary material

Supplementary figure legends

Supplementary figures

## References

[1] Torre LA (2015). Global cancer statistics, 2012. CA: Cancer J. Clin..

[2] Campisi J (2001). Cellular senescence as a tumor-suppressor mechanism. Trends Cell Biol..

[3] Braig M (2005). Oncogene-induced senescence as an initial barrier in lymphoma development. Nature.

[4] Chen Z (2005). Crucial role of p53-dependent cellular senescence in suppression of Pten-deficient tumorigenesis. Nature.

[5] Collado M (2005). Tumour biology: senescence in premalignant tumours. Nature.

[6] Collado M, Serrano M (2006). The power and the promise of oncogene-induced senescence markers. Nat. Rev. Cancer.

[7] Collado M, Blasco MA, Serrano M (2007). Cellular senescence in cancer and aging. Cell.

[8] Acosta JC, Gil J (2012). Senescence: a new weapon for cancer therapy. Trends Cell Biol..

[9] Xue W (2007). Senescence and tumour clearance is triggered by p53 restoration in murine liver carcinomas. Nature.

[10] Eggert T (2016). Distinct Functions of Senescence-Associated Immune Responses in Liver Tumor Surveillance and Tumor Progression. Cancer Cell.

[11] Beier F, Martinez P, Blasco MA (2015). Chronic replicative stress induced by CCl4 in TRF1 knockout mice recapitulates the origin of large liver cell changes. J. Hepatol..

[12] Tanguay RM (1990). Different molecular basis for fumarylacetoacetate hydrolase deficiency in the two clinical forms of hereditary tyrosinemia (type I). Am. J. Hum. Genet.

[13] van Spronsen FJ (1994). Hereditary tyrosinemia type I: a new clinical classification with difference in prognosis on dietary treatment. Hepatology.

[14] van Spronsen FJ, Bijleveld CM, van Maldegem BT, Wijburg FA (2005). Hepatocellular carcinoma in hereditary tyrosinemia type I despite 2-(2 nitro-4-3 trifluoro- methylbenzoyl)-1, 3-cyclohexanedione treatment. J. Pediatr. Gastroenterol. Nutr..

[15] Wangensteen KJ (2008). A facile method for somatic, lifelong manipulation of multiple genes in the mouse liver. Hepatology.

[16] Xiang D (2014). Non-viral FoxM1 gene delivery to hepatocytes enhances liver repopulation. Cell Death Dis..

[17] Huang P (2011). Induction of functional hepatocyte-like cells from mouse fibroblasts by defined factors. Nature.

[18] Yu B (2013). Reprogramming fibroblasts into bipotential hepatic stem cells by defined factors. Cell Stem Cell.

[19] Huang P (2014). Direct reprogramming of human fibroblasts to functional and expandable hepatocytes. Cell Stem Cell.

[20] Li F (2010). Hepatoblast-like progenitor cells derived from embryonic stem cells can repopulate livers of mice. Gastroenterology.

[21] Orejuela D, Jorquera R, Bergeron A, Finegold MJ, Tanguay RM (2008). Hepatic stress in hereditary tyrosinemia type 1 (HT1) activates the AKT survival pathway in the fah^−/−^ knockout mice model. J. Hepatol..

[22] Endig J (2016). Dual role of the adaptive immune system in liver injury and hepatocellular carcinoma development. Cancer Cell.

[23] Aravinthan AD, Alexander GJ (2016). Senescence in chronic liver disease: Is the future in aging?. J. Hepatol..

[24] Grompe M (1993). Loss of fumarylacetoacetate hydrolase is responsible for the neonatal hepatic dysfunction phenotype of lethal albino mice. Genes Dev..

[25] Larochelle J (2012). Effect of nitisinone (NTBC) treatment on the clinical course of hepatorenal tyrosinemia in Quebec. Mol. Genet. Metab..

[26] Grompe M (1995). Pharmacological correction of neonatal lethal hepatic dysfunction in a murine model of hereditary tyrosinaemia type I. Nat. Genet.

[27] Buitrago-Molina LE (2013). The degree of liver injury determines the role of p21 in liver regeneration and hepatocarcinogenesis in mice. Hepatology.

[28] Hayflick L (1965). The limited in vitro lifetime of human diploid cell strains. Exp. Cell Res..

[29] Willenbring H (2008). Loss of p21 permits carcinogenesis from chronically damaged liver and kidney epithelial cells despite unchecked apoptosis. Cancer Cell.

[30] Vogel A (2004). Chronic liver disease in murine hereditary tyrosinemia type 1 induces resistance to cell death. Hepatology.

[31] Wang MJ (2014). Reversal of hepatocyte senescence after continuous in vivo cell proliferation. Hepatology.

[32] Dimri GP (1995). A biomarker that identifies senescent human cells in culture and in aging skin in vivo. Proc. Natl Acad. Sci. USA.

[33] Lee BY (2006). Senescence-associated beta-galactosidase is lysosomal beta-galactosidase. Aging Cell.

[34] Campisi J (2013). Aging, cellular senescence, and cancer. Annu. Rev. Physiol..

[35] Lowe SW, Cepero E, Evan G (2004). Intrinsic tumour suppression. Nature.

[36] Stuart GR, Glickman BW (2000). Through a glass, darkly: reflections of mutation from lacI transgenic mice. Genetics.

[37] Stocker E, Heine WD (1971). Regeneration of liver parenchyma under normal and pathological conditions. Beitr. Pathol..

[38] Agarwal R, Narayan J, Bhattacharyya A, Saraswat M, Tomar AK (2017). Gene expression profiling, pathway analysis and subtype classification reveal molecular heterogeneity in hepatocellular carcinoma and suggest subtype specific therapeutic targets. Cancer Genet.

[39] Zhang, Y. L. et al. SPON2 Promotes M1-like macrophage recruitment and inhibits hepatocellular carcinoma metastasis by distinct integrin-rho GTPase-Hippo Pathways. Preprint at https://cancerres.aacrjournals.org/content/early/2018/02/13/0008-5472.can-17-2867# (2018). 10.1158/0008-5472.CAN-17-2867 29440144

[40] He Wei, Gao XM, Cao XT, Xiong. SD (2008). Medical Immunology.

[41] Kang TW (2011). Senescence surveillance of pre-malignant hepatocytes limits liver cancer development. Nature.

[42] Dou Z (2017). Cytoplasmic chromatin triggers inflammation in senescence and cancer. Nature.

[43] Wangensteen, K. J. et al. Combinatorial genetics in liver repopulation and carcinogenesis with a novel in vivo CRISPR activation platform. Preprint at https://aasldpubs.onlinelibrary.wiley.com/doi/abs/10.1002/hep.29626 (2017). 10.1002/hep.29626 PMC5930141 29091290

[44] R Development Core Team (2011), R: A Language and Environment for Statistical Computing. Vienna, Austria: the R Foundation for Statistical Computing. http://www.R-project.org/.

[45] Fan J (2016). Characterizing transcriptional heterogeneity through pathway and gene set overdispersion analysis. Nat. Methods.

[46] Shalapour S (2017). Inflammation-induced IgA + cells dismantle anti-liver cancer immunity. Nature.

