# Supplementary material for: The extent of liver injury determines hepatocyte fate toward senescence or cancer
Source: Cell Death Dis. 2018 May 14;9(5):575. doi: 10.1038/s41419-018-0622-x (PMC5951829; doi:10.1038/s41419-018-0622-x)
Supplement: Supplementary file 1 — Supplementary figure legends [file 41419_2018_622_MOESM1_ESM.doc]

**Supplementary Figure 1**

**Pathological changes of liver in** ***Fah^-/-^* mice under severe acute and** **moderate chronic liver injury.**

(A) The captured photographs show hematoxylin-eosin (H&E) staining the liver tissues of *Fah^-/-^* mice under SALI or MCLI at 0, 4 and 8 weeks. (a, b) Representative parts of photographs are enlarged to indicate the views of corresponding positions. (B) Oil red O staining for sections of liver samples from *Fah^-/-^* mice under SALI or MCLI at 0 and 8 weeks. (C) Serum indexes (TC, TG) of blood samples from *Fah^-/-^* mice under SALI or MCLI at 0 and 8 weeks. (D-F) The bar graphs show the quantification of hepatocytes size in the livers of *Fah^-/-^* mice under SALI or MCLI at 0, 4 and 8 weeks. TG: triglyceride, TC: total cholesterol. All values presented as mean ± S.D. ***p* < 0.01. Scale bar, 100 µm.

**Supplementary Figure 2**

**Serum indexes and** **TUNEL assay in livers of *Fah^-/-^* mice under severe acute and moderate chronic liver injuries.**

(A) Serum indexes of liver function parameters (ALT, AST, and T-bil) from blood samples of *Fah^-/-^*mice under SALI or MCLI at 0, 4 and 8 weeks. (B) The representative photographs show TUNEL assay on the liver tissues of *Fah^-/-^* mice under SALI or MCLI at 0, 4 and 8 weeks. (C) The bar graphs show the quantification of TUNEL-positive hepatocytes in the liver samples of *Fah^-/-^* mice under SALI or MCLI at 0, 4 and 8 weeks. AST: aspartate aminotransferase, ALT: alanine aminotransferase, T-bil: total bilirubin. All values presented as mean ± S.D. ***p* < 0.01.

**Supplementary Figure 3**

**Statistical chart of body weight in *Fah^-/-^* mice under severe acute and moderate chronic liver injury.**

The arrow indicates the addition of NTBC.
