# Supplementary material for: The extent of liver injury determines hepatocyte fate toward senescence or cancer
Source: Cell Death Dis. 2018 May 14;9(5):575. doi: 10.1038/s41419-018-0622-x (PMC5951829; doi:10.1038/s41419-018-0622-x)
Supplement: Supplementary file 2 — Supplementary figures [file 41419_2018_622_MOESM2_ESM.doc]

**Supplementary Figures**

**Supplementary Figure 1**

**
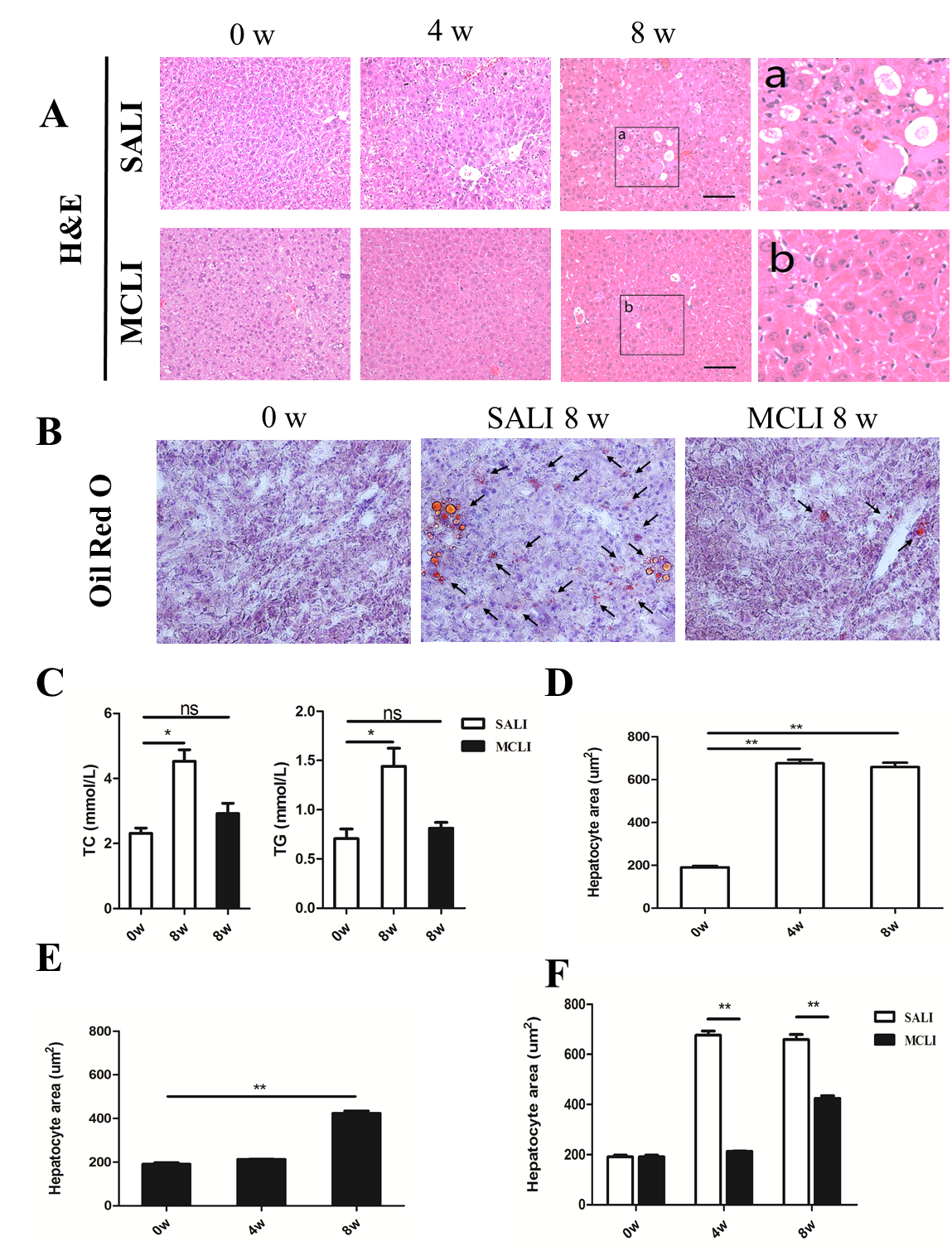
**

**Supplementary Figure 2**

**
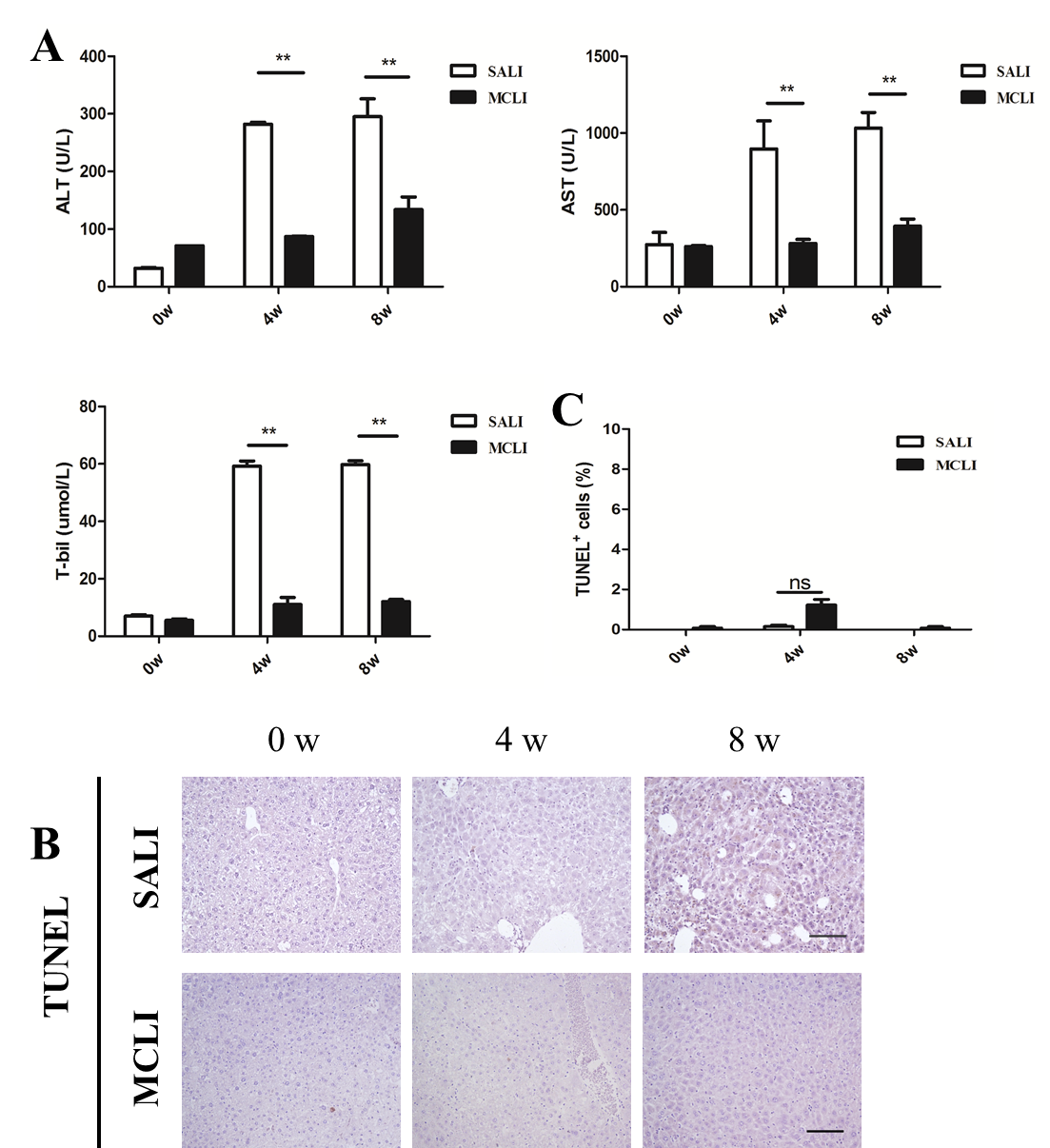
**

**Supplementary Figure 3**

**
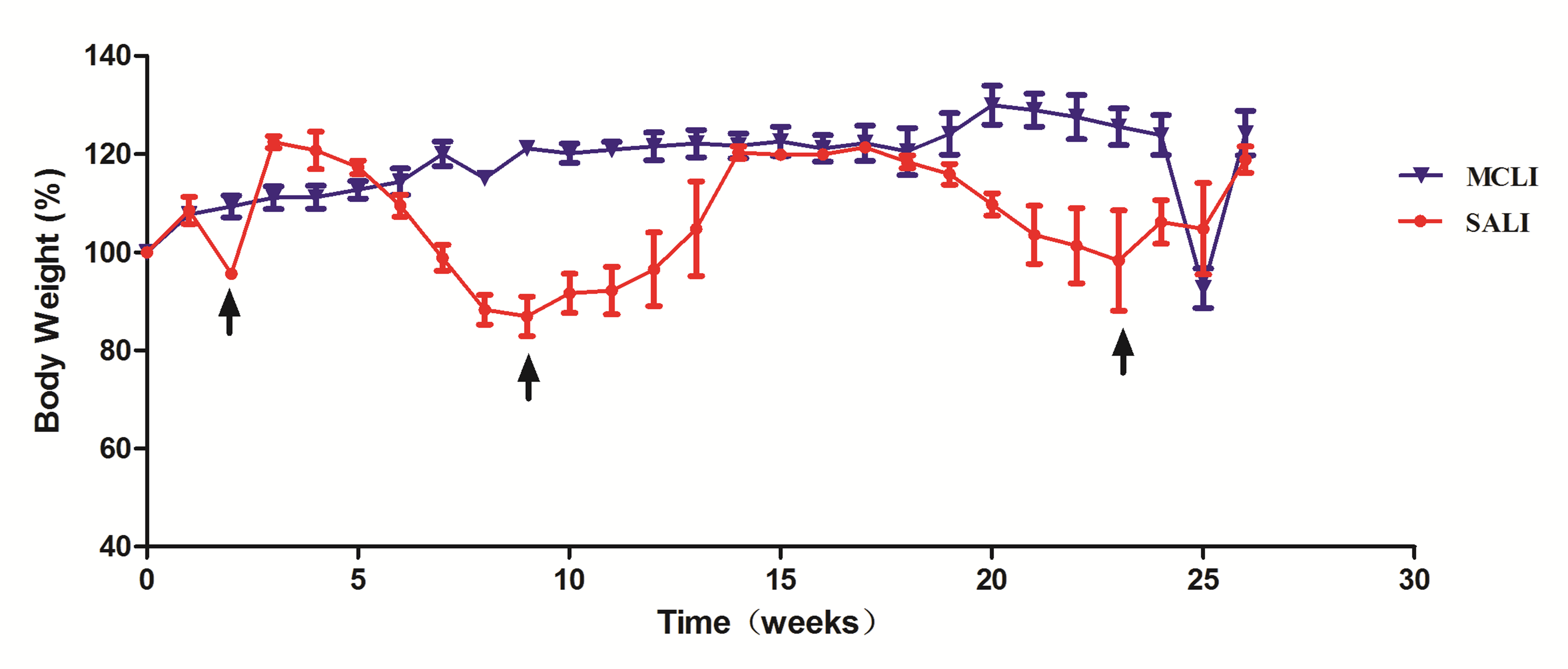
**

**Supplementary Table 1**

Primary antibodies used in the IHC and western blot assay

| Primary Antibody | Brand | Cat. No. | | Dilution ratio | application |
| --- | --- | --- | --- | --- | --- |
| P16 | Santa Cruz | sc-74401 | 1:500 | | IHC |
| P21 | Santa Cruz | sc-6246 | 1:1000 | | Western |
|  |  |  | 1:500 | | IHC |
| P53 | Abcam | ab26 | 1:1000 | | Western |
|  |  |  | 1:500 | | IHC |
| p-Rb | Cell Signaling | 9308 | 1:1000 | | Western |
|  |  |  | 1:500 | | IHC |
| CDK4 | Proteintech | 11026-1-AP | 1:2000 | | Western |
|  |  |  | 1:1000 | | Western |
| CDK2 | Proteintech | 10122-1-AP | 1:1000 | | Western |
| CDK6 | Proteintech | 14052-1-AP | 1:1000 | | Western |
| GAPDH | Proteintech | HRP-60004 | 1:6000 | | Western |
| Alpha tubulin | Abcam | ab15246 | 1:5000 | | Western |
| CD57 | Abcam | ab199156 | 1:500 | | IHC |
| T-bet | Abcam | ab91109 | 1:200 | | IHC |
| CD68 | Thermo Fisher | MA5-13324 | 1:100 | | IHC |
| CCL2 | Proteintech | 66272-1-AP | 1:500 | | IHC |
| CD11b | Proteintech | 20991-1-AP | 1:500 | | IHC |

**Supplementary Table 2**

**Genes related to macrophage activations** enriched in cluster 1 after GO term enrichment analysis.

| **ID** | **Category** | **p value** | **Term** |
| --- | --- | --- | --- |
| 10890 | GO:0042116 | 0.00020261 | Macrophage activation |
| 11370 | GO:0043030 | 0.000888895 | Regulation of macrophage activation |
| 11372 | GO:0043032 | 0.001134345 | Positive regulation of macrophage activation |
| 4998 | GO:0010759 | 0.014540593 | Positive regulation of macrophage chemotaxis |
| 4997 | GO:0010758 | 0.027899751 | Regulation of macrophage chemotaxis |
| 10564 | GO:0036301 | 0.028970852 | Macrophage colony-stimulating factor production |
| 18791 | GO:1901256 | 0.028970852 | Regulation of macrophage colony-stimulating factor production |
| 13434 | GO:0048246 | 0.044451105 | Macrophage chemotaxis |
